# Generalized framework for identifying meaningful heterogenous treatment effects in observational studies: A parametric data-adaptive G-computation approach

**DOI:** 10.1177/09622802251316969

**Published:** 2025-02-24

**Authors:** Roch A. Nianogo, Stephen O’Neill, Kosuke Inoue

**Affiliations:** 1 Department of Epidemiology, Fielding School of Public Health, University of California, Los Angeles (UCLA), USA; 2 California Center for Population Research, University of California, Los Angeles (UCLA), USA; 3 Department of Health Services Research and Policy, London School of Hygiene and Tropical Medicine, UK; 4 Department of Social Epidemiology, Graduate School of Medicine, 12918 Kyoto University, Japan; 5 Hakubi Center, 12918 Kyoto University, Japan

**Keywords:** Heterogeneity, effect modification, clustering, machine learning, regularization, causal inference, explanation, epidemiology

## Abstract

There has been a renewed interest in identifying heterogenous treatment effects (HTEs) to guide personalized medicine. The objective was to illustrate the use of a step-by-step transparent parametric data-adaptive approach (the generalized HTE approach) based on the G-computation algorithm to detect heterogenous subgroups and estimate meaningful conditional average treatment effects (CATE). The following seven steps implement the generalized HTE approach: Step 1: Select variables that satisfy the backdoor criterion and potential effect modifiers; Step 2: Specify a flexible saturated model including potential confounders and effect modifiers; Step 3: Apply a selection method to reduce overfitting; Step 4: Predict potential outcomes under treatment and no treatment; Step 5: Contrast the potential outcomes for each individual; Step 6: Fit cluster modeling to identify potential effect modifiers; Step 7: Estimate subgroup CATEs. We illustrated the use of this approach using simulated and real data. Our generalized HTE approach successfully identified HTEs and subgroups defined by all effect modifiers using simulated and real data. Our study illustrates that it is feasible to use a step-by-step parametric and transparent data-adaptive approach to detect effect modifiers and identify meaningful HTEs in an observational setting. This approach should be more appealing to epidemiologists interested in explanation.

## 1 Introduction

In public health and clinical settings, it is not uncommon to observe some heterogeneity in intervention or treatment effects—which occurs when the effect of an intervention or treatment is different from one subgroup to another.^1–3^ Epidemiologists have been long interested in investigating heterogenous treatment effects (HTEs) for several reasons.^1,2,4^ First, it is well-known that one-size-fits-all treatments across individuals could lead to disparate benefits and harms in some groups. For instance, statins are not recommended for all patients but only for those with a certain risk factor profile—this is to maximize benefit among those at risk and limit the harms in subgroups less at risk.^5^ Second, tailoring interventions or policies and targeting subgroups who are most at risk or who would benefit the most given limited resources could potentially lead to the greatest societal impact.^6–9^ This is particularly relevant in disparities research as the detection of differences in treatment effects across socio-economic or racial/ethnic subgroups could help identify potential barriers and levers for achieving optimal health for all, as failing to identify such barriers could lead to unavoidable deaths and exacerbate existing disparities.^10–15^ More recently, there has been an increased interest in identifying HTE to guide personalized medicine^16–18^ as well as to further investigate negative trials, that is, those that report a null total effect, as it could reveal the presence of treatment benefit for some subgroups (e.g. those whose body mass index is ≥30 kg/m^2^) but not all.^19–21^

### 1.1 Brief summary of conventional versus modern approaches to detecting heterogeneity

The identification of HTEs, also known as effect modification or moderation is not a novel concept in epidemiology,^3,22^ however, its recent application in personalized interventions has given researchers a renewed appreciation for its importance. Conventional methods have long made use of statistical interactions and parametric models to detect heterogeneity.^3,22^ Over the past decade, many tools designed to detect HTEs have emerged across disciplines such as computer science, statistics, econometrics, and epidemiology. These innovations have yielded “non-parametric” estimators and have introduced techniques to detect HTEs leveraging data-adaptive (machine learning) algorithms. Many of these approaches for estimating individual-level effects belong to the family of ‘meta-learner’ approaches^23^ in that they either: (a) estimate a single model for all units [S-learners], (b) estimate separate models for treated and control units [T-learners], (c) model the propensity for treatment as well as modelling outcomes [X-learners] or (d) ‘orthogonalize’ outcomes and treatment assignment prior to estimation (that is, they use residuals from models in place of observed variables) [R-learners]. For example, causal forest, a commonly used R-learner approach^24–28^ uses an ensemble of trees optimized to detect HTEs, allowing us to estimate Conditional Average Treatment Effect (CATE) for each individual as a function of multi-dimensional individual characteristics. Causal forests differ from random forests in that they predict the treatment effect (e.g. outcome improvement due to the treatment) for each individual and seek to identify its variations, whereas random forests primarily focus on predicting the outcome itself. To reduce the risk of overfitting, causal forests also employ an approach, called “honest estimation” when building each decision tree, ensuring an observation is not simultaneously used to determine sample splits and to estimate effects.

### 1.2 Three challenges when considering machine learning or conventional approaches to detect HTEs and estimate subgroup effects

When opting for novel machine learning (e.g. causal forest) or conventional approaches (e.g. parametric models with interaction terms) to detect HTEs and estimate subgroup effects, three potential challenges are at the forefront of the epidemiologist's mind: (a) the problem of accuracy versus interpretability, (b) the problem of underfitting or overfitting when using parametric models, and (c) the problem of type 1 error and joint testing.

#### 1.2.1 The problem of accuracy versus interpretability

Many non-parametric machine-learning algorithms such as neural networks and random forests are excellent in producing accurate predictions; however, this often comes at the cost of model interpretability, since they are highly non-linear (neural networks) or average across many decision trees (random forests), making them somewhat of a ‘black box’ where the relationship between inputs and outputted predictions is opaque. This has led to a growing literature on interpretability and explainable machine-learning modeling.^29^ On the other hand, traditional parametric linear or logistic models while highly interpretable can produce poor prediction accuracy. This phenomenon known as the “two cultures” is hardly a novel problem and has been well described by Breiman (2001) who lamented the underuse of machine learning over conventional parametric modeling for solving prediction problems.^30^ With more recent advances in both “cultures”, it is conceivable to envision a scenario where both approaches/cultures are used to produce models with good enough prediction accuracy and acceptable interpretability. One such example is that of regularization methods (e.g. ridge regression) which are class of “interpretable”^31^ parametric machine-learning algorithms that do not sacrifice too much interpretability but with much-improved performance accuracy.

Furthermore, selecting an adequate modeling approach is particularly relevant when one is interested in an explanation/causal inference problem as opposed to a purely predictive problem per se.^31^ In fact, knowledge about the underlying data-generating process or causal structural model is crucial in guiding the inclusion of variables in one's model when the goal is to elucidate the causal relationship between exposure and outcome.^32^ The investigator would include variables sufficient for controlling confounding in their model while excluding descendants of the primary exposure of interest, such as colliders.^33–35^

#### 1.2.2 The problem of underfitting or overfitting when using parametric modeling

Underfitting occurs when a model is not sufficiently rich to capture the true relationship between covariates and outcomes, while overfitting occurs when the model is too flexible capturing idiosyncratic variation and leading to poor out-of-sample performance. Thus, there is a bias variance trade-off when specifying models. Data-driven machine-learning approaches assess model performance out of sample allowing these risks to be balanced. It has been recognized that parametric regressions can achieve similar estimates to non-parametric, data-driven approaches, when models are flexibly specified allowing for rich interactions between covariates and treatment^3^ but at the cost of overfitting.^36^

For prediction tasks, one could fit a saturated parametric model with reasonable n-way interaction terms and then use a shrinkage-based method to correct for the presence of unnecessary variables such as by shrinking their coefficient toward zero. Such methods include regularization methods like Ridge regression, Least Absolute Shrinkage and Selection Operator (LASSO),^37^ and Elastic Net models.^38^ The use of regularization methods has been shown to be superior to the ordinary least square (OLS) model when the regularization hyperparameters are well-tuned such as through cross-validation.^38^

Where interest centers on estimating effects in observational settings, a post-double selection approach,^39^ where variables that are selected as being predictive of outcomes or treatment assignment are chosen for inclusion can also be used to reduce bias. For subgroup analysis, consideration of variables predictive of treatment-covariate interactions may also be necessary.^40^

Additional approaches can include the use of inverse probability of treatment weights (IPTW) for adjusting for residual confounding.^41^

#### 1.2.3 The problem of type 1 error and joint testing^42,43^

After estimating individual-level treatment effects, one is often interested in aggregating these for subgroups of interest. The most-principled approach is arguably to pre-specify the groups for which effects will be presented,^44^ while data-adaptive approaches to identify subgroups have also been proposed.^45–51^ A number of studies investigating HTEs when using machine-learning methods report the effect of the exposure on the outcome across subgroups defined by combinations of covariates and oftentimes further report such effects across all covariates in the model.^21,36^ Reporting the effects across all covariates and then highlighting the ones that are “significant” can be seen at best as a hypothesis-generating endeavor rather than confirmatory^52^ and at worst as a form of significance chasing. The latter can lead to false positives^53^ and threats of type 1 error,^54,55^ particularly when multiple testing adjustments (e.g. Bonferroni correction, Semi-Bayes,^56–58^ Benjamini-Hochberg) are not accounted for.

In particular, our proposed approach deals with type 1 error in the following manner. First, it employs regularization methods such as LASSO^37^ or Elastic Net models^38^ which effectively performs variable selection by shrinking less important coefficients to zero. This reduces the number of hypotheses tested and controls Type I error by penalizing the inclusion of non-informative variables. Second, cross-validation helps in assessing the model's performance and increases its generalization capability. This reduces the risk of Type I errors by ensuring that the model's performance is not overly optimistic due to overfitting. Third, achieving balance in covariates between candidate subgroups can help prevent the false discovery of HTE subgroups as demonstrated by Rigdon et al.^42^ Fourth, our approach helps reduce the risk of type I error by only evaluating effects across the subgroups and variables that have been (pre)-identified as potential effect modifiers by our generalized HTE approach.

In the current paper, we describe the use of a theory- and data-driven approach to detect meaningful HTEs (*the generalized HTE approach*) and estimate subgroup treatment effects using the G-computation algorithm,^59^ while recognizing these 3 challenges.

**Box.** Definitions

1. **Effect modifier/moderator**: a variable that differentially modifies the observed effect of the intervention on the outcome.
2. **Average Treatment Effect (ATE)**: the expected effect of an intervention in the total population, that is *τ* = *E*(*Y*^1^ − *Y*^0^)
3. **Heterogenous Treatment Effects (HTE)**: difference in effects of an intervention across individuals or groups.
4. **Conditional Average Treatment Effects (CATEs)**: the expected effect for individuals with a particular covariate profile (e.g. *W = w*), that is, a contrast of the *expected* potential outcomes for that individual under each treatment: *τ*(*w*) = *E*(*Y*^1^ − *Y*^0^|*W* = *w*)*.* It is also sometimes referred to as individualized treatment effect (ITE) when each individual is defined by the values of *W*.
5. **Subgroup Average Treatment Effects**: the average effect for individuals in a particular group (e.g. *G = g*), that is *τ*(*g*) = *E*(*Y*^1^ − *Y*^0^|*G* = *g*)

## 2 Methods

In implementing the *generalized HTE approach*, we make use of a parametric “interpretable” supervised machine-learning algorithm using regularization methods such as LASSO and a split sample approach (i.e. using the training subsample for model development and test subsample for making model prediction) to prevent overfitting and improve the generalization performance of the model. We also use an unsupervised machine-learning algorithm such as clustering to identify effect modifiers. We then obtain effect estimates using G-computation (also referred to as recycled predictions^60,61^ or direct standardization) by contrasting estimated potential outcomes under each treatment. G-computation is a generalization of the standardization method for time-varying settings that has been used in the epidemiologic literature to (a) obtain standardized estimate over all covariates and interactions,^62–64^ (b) adjust for time-varying confounders,^65–68^ and (c) to project the impact of hypothetical interventions.^69–72^

### 2.1 Notations and definitions

Let *Y* be the outcome, *X* the exposure or treatment, *Y^X^*^=^*^x^* the potential outcome *Y* under treatment *X* = *x, C* a set of confounders sufficient for confounding control and *W* a set of potential effect modifiers.

### 2.2 Identifiability conditions

We assume conditional exchangeability (unconfoundedness or ignorability) given confounders, *C* within *W* = *w*. In other words, the potential outcome *Y^X^* had treatment *X* been set to *x*, is independent of treatment *X* given adjusted confounding variables *C* within *W* = *w*; where *C* satisfies the backdoor criterion within *W = w*,^73,74^ that is, contains no descendants of *X* (e.g. colliders) and that upon conditioning on *C*, there is no open backdoor path between *X* and *Y* within *W* = *w*. Importantly, the confounder set *C* includes all potential modifiers, *W* that are also confounders (i.e. when *C* and *W* are not mutually exclusive and there exist covariates that are in both *C* and *W*). Other assumptions include positivity (common support), consistency (treatment irrelevance),^75^ no interference or SUTVA (stable unit treatment value assumption),^76^ no model functional form misspecifications, no measurement error and no selection bias.

### 2.3 Estimation of conditional average treatment effects

When the aforementioned identifiability conditions are satisfied, we can estimate the CATE using observational data. More formally, CATE can be derived from the following equation.

$$\begin{array}{ll} \text{CATE} & =\tau (w)=E(Y^{X=1}-Y^{X=0}|W=w)\\ & =E(Y^{X=1}|\;W=w)-E(Y^{X=0}|\;W=w)\\ & =\underset{C=c}{\sum }\;E(Y|X=1,\;W=w,C=c)\;P(C=c|W=w)\\ & \;-\underset{C=c}{\sum }\;E(Y|X=0,\;W=w,C=c)\;P(C=c|W=w) \end{array}$$

To date, several non-parametric algorithms such as meta-learners including causal forests have been used to estimate CATE. Nevertheless, the factors influencing the estimated effects obtained using these non-parametric approaches may not always be as transparent or readily interpretable as those from conventional parametric models. Therefore, in the subsequent section, we delineate each step of a parametric approach, *the generalized HTE approach*, to detect meaningful HTEs and estimate CATE.

### 2.4 G-computation steps in identifying HTE and estimating CATE

#### 2.4.1 Step 1: select variables that satisfy the backdoor criterion and potential effect modifiers

In this essential step, the investigator identifies the set of covariates sufficient for confounding control that satisfy the backdoor criterion,^74^ and selects potential effect modifiers with particular care taken to remove potential colliders (to avoid collider-stratification bias^33^). This can be aided by the use of directed acyclic diagrams^34^ and background knowledge^32^ which can be particularly important when using data-driven approaches for variable selection.^77^

#### 2.4.2 Step 2: specify a flexible saturated model including potential confounders and potential effect modifiers

An appropriate parametric model should be specified (e.g. Gaussian, Binomial). To capture heterogeneity, the investigator could specify a saturated model, that is a model that includes all possible n-way interaction terms between the treatment indicator and other included variables.

#### 2.4.3 Step 3: apply a selection method to reduce overfitting

This step can occur simultaneously with the previous step. Here, regularization methods such as Ridge regression, LASSO or elastic net could be used to reduce overfitting.

LASSO aims to find the set of coefficients that minimizes the sum-of-squared errors subject to a constraint on the sum of absolute values of coefficients. A penalty function is added to the typical Ordinary Least Squares (OLS) loss function, as follows:

$$\text{Loss}=\;\overset{N}{\underset{i=1}{\sum }}\;{\left(Y_{i}-\overset{J}{\underset{j=1}{\sum }}\;{\hat{\beta }}_{j}c_{ij}\right)}^{2}+\lambda \overset{J}{\underset{j=1}{\sum }}\;|{\hat{\beta }}_{j}|$$

where *Y* represents the dependent variable, *c_ij_* is the *j*th of *J* (rescaled) covariates for individual *i* and *β_j_* is the corresponding coefficient. The tuning parameter, *λ*, determines the extent to which the model complexity is penalized, with larger values resulting in more variables being excluded.

In contrast, and for illustration purposes, Ridge regression, shrinks coefficients toward 0, with smaller coefficients shrunk more but unlike LASSO, does not exclude variables.

$$\text{Loss}=\overset{N}{\underset{i=1}{\sum }}\;{\left(Y_{i}-\overset{J}{\underset{j=1}{\sum }}\;{\hat{\beta }}_{j}c_{ij}\right)}^{2}+\lambda \overset{J}{\underset{j=1}{\sum }}\;{{\hat{\beta }}_{j}}^{2}$$

For Elastic Net, the loss function to be minimized is:

$$\text{Loss}=\overset{N}{\underset{i=1}{\sum }}\;{\left(Y_{i}-\overset{J}{\underset{j=1}{\sum }}\;{\hat{\beta }}_{j}c_{ij}\right)}^{2}+\lambda \left(\frac{1-\alpha }{2}\overset{J}{\underset{j=1}{\sum }}\;{\hat{\beta }}_{j}^{2}+\alpha \overset{J}{\underset{j=1}{\sum }}\;|{\hat{\beta }}_{j}|\right)$$

Note that when α = 1, elastic net is equivalent to LASSO, while when α → 0, elastic net approaches ridge regression. When *λ* = 0, the penalty function corresponds to the typical OLS loss function.

In effect, Elastic Net models are types of more transparent parametric supervised machine-learning algorithms that simultaneously allow for model selection, the shrinking of some coefficients toward zero (e.g. Ridge regression) and even the setting of some coefficients to zero (e.g. LASSO). This allows for a sparse and parsimonious model and avoids overfitting. In fact, the goal of these regularization methods is to avoid overfitting by adding some penalization for each additional term in the model. This step requires, however, the tuning of hyperparameters (lambda and alpha), typically done through cross-validation.

While the above approaches can be readily applied when interest is on avoiding overfitting in predictions, when interest is in estimating causal effect, these approaches may not adequately control for potential confounders in some cases (e.g. when confounders are selected out of the model). Including additional variable selection steps that identify variables influencing both treatment decisions and outcome and aided by background knowledge,^32^ can be beneficial. Re-estimating the outcome model using the union of variables selected can mitigate this concern and provide valid inference.^39^ This is also relevant when the interest is in effect modification as performing variable selection on interactions between modifiers and treatment is also important and recommended,^40^ albeit it may increase the risk of selecting bad controls,^77^ so care must be taken to consider the appropriateness of selected covariates. The additional variable selection step is known as the post-double selection^39^ and is done on the training sample.

Additionally, as interests are in causal effects, one can instead use IPTW to adjust for residual confounding,^41^ and achieve balance.

#### 2.4.4 Step 4: predict potential outcomes under treatment and no treatment

Using the fitted model developed in the training data (with the hyperparameters selected in the model development in step 3), we can then predict potential outcomes under treatment, ${\hat{Y}}_{i}^{X=1}$ , and under no treatment, ${\hat{Y}}_{i}^{X=0}$ , in the testing/validation data.

#### 2.4.5 Step 5: obtain a contrast between potential outcomes for each individual

The contrast between the potential outcomes under the different treatment scenarios can be expressed for instance as mean difference (MD; a difference between means) or risk differences (RDs; difference between predicted probabilities). Such individual-level CATE estimates are conditioned on each value of individual's characteristics and are sometimes called individualized treatment effects (ITE).

#### 2.4.6 Step 6: fit cluster modeling to find appropriate clusters of effects and identify potential effect modifiers

Once individual-level CATE estimates are obtained, they can be aggregated to obtain subgroup effects. This endeavor can be aided by using clustering algorithms, which are a type of unsupervised machine-learning technique that are helpful in finding subgroups of similar individuals. This machine-learning procedure avoids having to find groups manually, reducing the risk of data-mining. It systematically and iteratively uses an algorithm to calculate the distance between each formed cluster. It then aims at reducing the distance within a subgroup and increasing the distance between groups. The k-means cluster algorithm, for instance, aims to partition *N* observations into *k* clusters/groups (*g*_1_, … , *g_k_*) such that the squared Euclidean distance between the row vector for any individual's variables (effect modifiers here) (*m_i_*) and the centroid vector of their respective cluster is at least as small as the distance to the centroids of the remaining clusters^78^ that is, they are assigned to the cluster they are most similar to in terms of the variables in *m*. An iterative procedure is used to determine the centroids and the cluster membership.^78^ Another similar clustering approach include the Ward hierarchical clustering^79^ which minimizes the total within-cluster variance aiming at minimizing the Euclidian distance.

Clustering can be used to identify potential effect modifiers. This is done using the following sub-steps. First, once individual CATEs have been estimated, a clustering algorithm can be used to identify clusters (typically but not always, two clusters are identified) in which individuals differ as much as possible between clusters (in terms of estimated effects here). These clusters are akin to the leaf nodes of a decision tree. Of note, some algorithms such as *k*-means or Ward clustering need to have a large number of distinct values that is greater than the number of clusters to be searched. Here, we can use a statistical trick by adding some small random noise (called jittering) to each CATE to help identify clusters. Jittering has been used in machine-learning model training and has been shown to help with model generalization.^80,81^ Note that this trick is only for identifying the best/optimal number of clusters. Second, each covariate *z*-scores across the identified clusters are estimated. Third, the variable importance calculated as the difference in covariate *z*-scores across clusters is also estimated. Selected potential effect modifiers are those whose variable importance exceeds a certain threshold, for instance, we use 0.2 in our illustration.

#### 2.4.7 Step 7: estimate subgroup CATEs in a marginal structural model

To estimate subgroup CATEs, we can include interaction terms between effect modifier(s) identified in Step 5 and the treatment in an OLS regression using the entire dataset. The subgroup CATEs are then simply obtained by finding the treatment effect across the subgroup defined by the identified effect modifiers in a marginal structural model (e.g. using G-computation or IPTW). If IPTW is used, robust sandwich estimators are used to estimate standard errors and 95% confidence intervals (CIs) and if G-computation, then bootstrap is used instead. Other estimators could also be used including propensity score matching (e.g. Ridge matching)^82^ doubly robust estimation,^83^ and other semiparametric estimators of the ATE (*Busso M, DiNardo J, McCrary J. Finite Sample Properties of Semiparametric Estimators of Average Treatment Effects, 2009, Unpublished*).

## 3 Illustration

As a proof of concept, we conducted two studies. First, we conduct a simulation study to assess the ability of *the generalized HTE approach* to detect HTEs and estimate CATEs. Second, we implement the method in an applied study using the Health and Retirement Study (HRS)^84^ to identify potential effect modifiers of the association between baseline low kidney function (as measured by an estimated glomerular filtration, eGFR) and incident dementia.

We next describe the implementation of the methods for both studies. We first fit a LASSO model on the training data (a random 70% of the data) to select relevant variables and interaction terms. The tuning parameters (lambda) were obtained via 10-fold cross-validation. We used inverse probability of treatment weighting (IPTW) to adjust for potential confounding. The fitted model was used on the testing data (the remaining 30%) to predict the potential outcomes under treatment and no treatment. We then took the contrast in terms of the difference between the potential outcomes to obtain the CATE estimates.

The Ward clustering approach^79^ which minimizes the total within-cluster variance was used to identify distinct clusters based on the CATE estimates. This approach minimized the Euclidian distance and selected the optimal number of clusters based on the Beale index. We identified potential effect modifiers by examining the variable importance calculated as the difference in covariate *z*-scores across clusters. In fact, selected potential effect modifiers were those whose variable importance was greater than 0.2. After identifying the potential effect modifiers, we fit a marginal structural model with robust standard error to estimate CATEs in the subgroups defined by the potential effect modifiers.

### 3.1 Application to simulated data

#### 3.1.1 Overview

In the simulation study, we employed a data-generating process in which we simulated an observational study inspired from the randomized controlled trial simulation study described in Rigdon et al.^42^ (see eSection 1 and eSection 2 for details on the data-generating mechanism and implementation of generalized HTE approach). We simulated 10,000 individuals (simulation A1) of whom about 30% were taking blood pressure medication to achieve a systolic blood pressure <120 mmHg to reduce cardiovascular outcomes^85^ (i.e., intensive blood pressure control) (see eTable 1 for baseline characteristics). To assess the method's capabilities, we also simulated different sizes (simulation A2, *N* = 100,000 and simulation A3, *N* = 1000). These three simulation studies used the same data-generating process. We report the results of simulation A1 in the main manuscript and appendix (Table 1, eFigure 1, eFigure 2, eFigure 3, eTable 2) and those of the simulation A2 and simulation A3 in the appendix only (eFigure 4 and eFigure 5).

**Table 1.** Subgroup CATE on the Additive and Relative Scale Presenting the Effect of Intensive Blood Pressure Control Treatment on Risk of a Cardiovascular Disease Event Across Moderators Identified Via the Generalized HTE Approach in the Simulated Data A1, *N* = 10,000.

Variables				*P for*	CATE risk	*P for*
and		Proportion	CATE risk difference	*interaction*	ratio (RR,	*interaction*
threshold	*N*	(%)	(RD, 95%CI)	*(RD)*	95%CI)	*(RR)*
Overall	100	10,000	−0.03 (−0.05, −0.02)	–	0.82 (0.75, 0.91)	–
eGFR < 73	51.59	5159	−0.12 (−0.14, −0.09)	0	0.51 (0.44, 0.59)	0
eGFR ≥ 73	48.41	4841	0.05 (0.03, 0.08)		1.37 (1.20, 1.56)	
Aspirin = 0	48.83	4883	−0.21 (−0.23, −0.19)	0	0.25 (0.21, 0.30)	0
Aspirin = 1	51.17	5117	0.13 (0.11, 0.16)		2.19 (1.93, 2.50)	

eGFR: estimated glomerular filtration rate; RD: risk difference, RR: risk ratio; CI: confidence interval.

### 3.2 Results

#### 3.2.1 Distribution of CATEs

Plotting the distribution of the CATEs (eFigure 1), we can see that the distribution is not unimodal but rather bimodal. In simulation A1, one of the distributions is centered toward a negative effect while the other around a positive effect. This preliminary step helps evaluate the potential for heterogeneity before identifying the presence of effect modifiers. This bi-model distribution can also be seen in a plot of CATE estimates by rank of CATE estimates (eFigure 2).

#### 3.2.2 Identifying potential effect modifiers

The variables whose importance exceeded a certain threshold, for example 0.2 were selected as potential effect modifier (eFigure 3).

In simulation A1 (*N* = 10,000), we identified two potential effect modifiers: aspirin use (a binary variable) and estimated glomerular filtration rate (eGFR, a continuous variable). Of note, the cut-off for binarizing eGFR was calculated as the average of the mean of eGFR across the two identified clusters. In our case, it was (75.31 + 70.74)/2 ≈ 73.0 (eTable 2). The other variables had a variable importance <0.2. Examining the variable importance plot (eFigure 3) suggests that the observed heterogeneity in eFigure 1 could potentially be due to the presence of effect modifiers.

#### 3.2.3 Estimating subgroup CATEs

In simulation A1, using the *generalized HTE approach*, we identified four meaningful subgroups defined by aspirin use or eGFR (Table 1): participants whose eGFR < 73 (RD: −0.12, 95%CI (−0.14, −0.09), participants whose eGFR ≥ 73 (RD: 0.05, 95%CI: 0.03, 0.08), participants not taking aspirin (RD: −0.21, 95%CI: −0.23, −0.19) and participants taking aspirin (RD: 0.13, 95%CI: 0.11, 0.16).

### 3.3 Application to real data

#### 3.3.1 Overview

For this application, we use 2006–2020 data from the US Health and Retirement Study (HRS), a longitudinal panel study that surveys a representative sample of about 20,000 Americans aged >50 years and their spouses. More information can be found here.^84^ In particular, we used the biomarker subsample data—a random 50% of the HRS data (in 2006 and 2008) which was preselected for the enhanced face-to-face interviews (EFTFI) which included collection of biomarker specimens (e.g. HbA1c). To obtain our analytical sample, we combined the 2006-subcohort with the 2008-subcohort; excluded individuals aged <50y and those with prevalent dementia at baseline in 2006/2008; implemented multiple imputation approach and used the first imputed dataset with complete data (*N* = 11,033, see Table 2 for baseline characteristics). It is known that low kidney function is positively associated with dementia.^86^ In the current illustration, the objective was to identify potential effect modifiers of the association between baseline low kidney function (as measured by an estimated glomerular filtration, eGFR) and incident dementia. More details on variable definition can be found in the appendix (eSection 3).

**Table 2.** Baseline Participants’ Characteristics in the Health and Retirement Student (HRS), *N* = 11,033.

Characteristics	Overall, *N* = 11,033	Low eGFR, *N* = 6519	Normal eGFR, *N* = 4514
Older age (Age ≥ 65), n (%)	6962 (63%)	6155 (94%)	807 (18%)
Female sex, n (%)	6500 (59%)	3766 (58%)	2734 (61%)
Participant years of education, Mean (SD)	12.7 (3.0)	12.5 (3.1)	13.0 (3.0)
Married, n (%)	7034 (64%)	3914 (60%)	3120 (69%)
Non-NH White participants, n (%)	2488 (23%)	1269 (19%)	1219 (27%)
Income < 130 of the FPL, n (%)	1329 (12%)	740 (11%)	589 (13%)
Low child SES, n (%)	5236 (47%)	3646 (56%)	1590 (35%)
APOE 4, n (%)	2930 (27%)	1690 (26%)	1240 (27%)
Systolic Blood Pressure (mmHg), Mean (SD)	131 (20)	134 (21)	128 (19)
HbA1c (%): NHANES Equivalent, Mean (SD)	5.87 (0.98)	5.88 (0.89)	5.86 (1.11)
HDL (mg/dL); NHANES Equivalent, Mean (SD)	54 (16)	54 (16)	55 (16)
BMI, kg/m^2^, Mean (SD)	29.4 (5.7)	28.6 (5.4)	30.4 (6.0)
Waist Circumference (cm), Mean (SD)	40 (6)	39 (6)	40 (6)
C-reactive Protein (mg/L); NHANES Equivalent, Mean (SD)	0.45 (0.87)	0.44 (0.92)	0.48 (0.78)
Pulse (Beats per Minute), Mean (SD)	70 (11)	69 (11)	72 (11)
Exercise (MET ≥ 35), n (%)	2598 (24%)	1401 (21%)	1197 (27%)
Never smoked, n (%)	6266 (57%)	3724 (57%)	2542 (56%)
Number of alcoholic drinks in last 3 months, Mean (SD)	0.69 (1.32)	0.58 (1.14)	0.85 (1.53)
CESD/Depression Score, Mean (SD)	1.39 (1.94)	1.28 (1.80)	1.55 (2.11)
Anxiety Index, Mean (SD)	1.57 (0.58)	1.55 (0.56)	1.60 (0.61)
State Anger Index, Mean (SD)	1.50 (0.50)	1.46 (0.48)	1.55 (0.53)
Trait Anger Index, Mean (SD)	2.18 (0.68)	2.13 (0.66)	2.26 (0.70)
Incident dementia, *n* (%)	1651 (15%)	1351 (21%)	300 (6.6%)

SD: Standard deviation; NH: not-Hispanic; SES: socio-economic status; APOE4: apolipoprotein E4 carrier; HbA1c: hemoglobin A1c; HDL: high-density lipoprotein; NHANES: National Health and Nutrition Examination Survey; MET: metabolic equivalent of task; CESD: Center for Epidemiologic Studies Depression Scale; FPL: federal poverty level; eGFR: estimated glomerular filtration rate. More details on variable definition can be found in the appendix (eSection 3).

### 3.4 Result

#### 3.4.1 Distribution of CATEs

**Figure 1.**
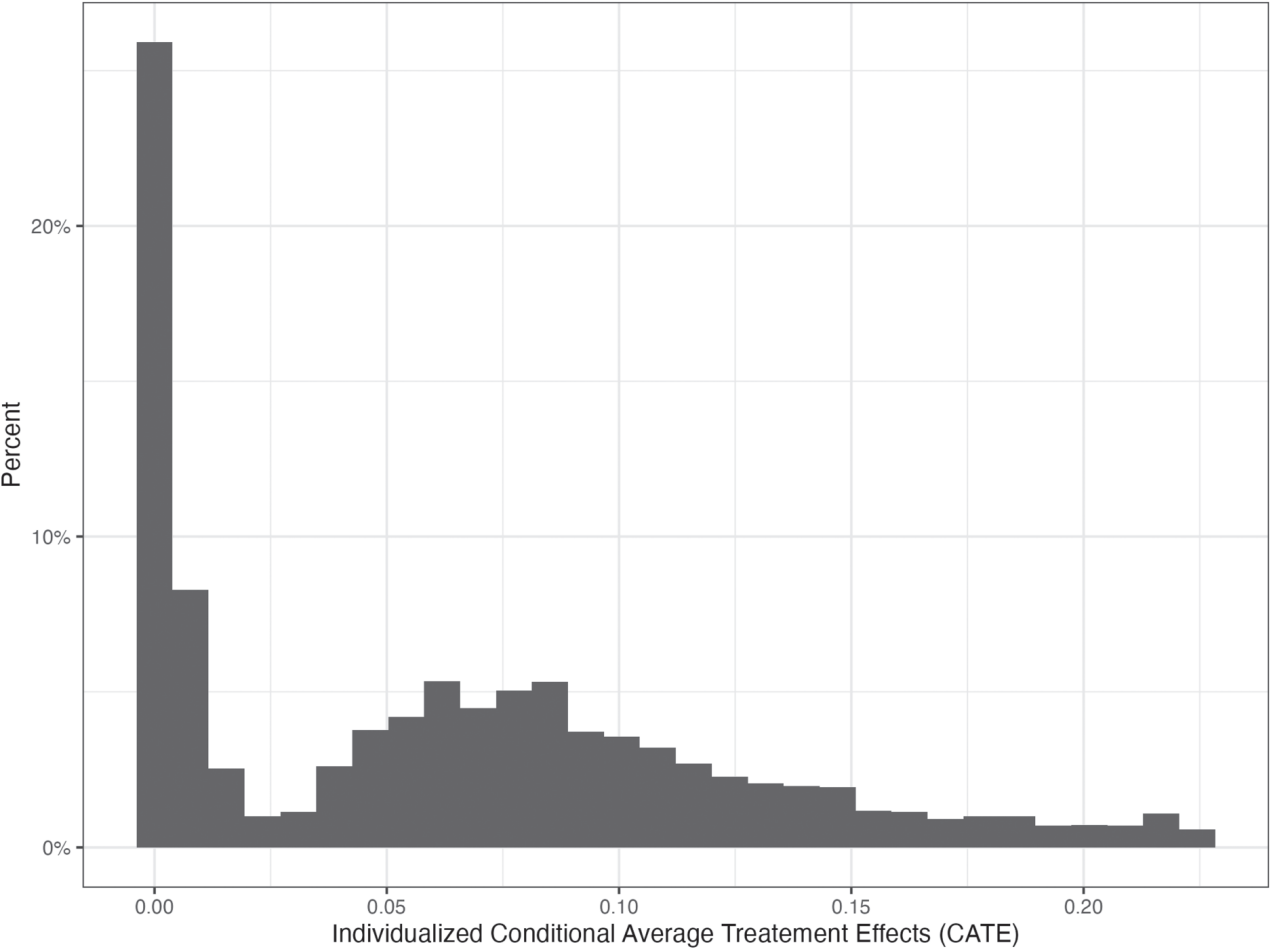
Histogram of the conditional average treatment effect (CATE) estimated via the generalized HTE approach in the health and retirement study (*N* = 11,033).

Plotting the distribution of the CATEs (Figure 1), we can see that the distribution is not unimodal but rather bimodal or made of at least two distinct distributions with different dispersion and central tendency parameters. One of the distributions is centered toward zero while the other is centered toward a larger effect. This mixed distribution can also be appreciated in a plot of CATE estimates by rank of CATE estimates (eFigure 6).

#### 3.4.2 Identifying potential effect modifiers

**Figure 2.**
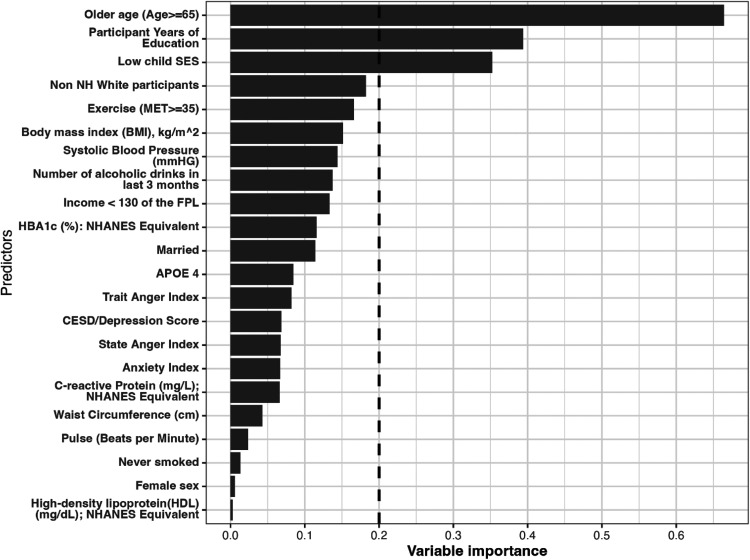
Variable importance estimated via the generalized HTE approach in the health and retirement study (*N* = 11,033). Only the top important covariates whose importance equal or exceed 0.20 were considered potential effect modifiers for the effect of treatment on the outcome.

As mentioned, the variables whose importance exceeded a certain threshold, for example 0.2 were included as potential effect modifier (Figure 2). In the HRS data, we identified three potential effect modifiers: older age (a binary variable, age ≥65 vs. <65), low childhood SES (a binary variable: low vs. high) and years of education (a continuous variable). For the continuous variable, the cut-off identified through the generalized HTE approach was calculated as the average of the years of education across the two identified clusters. In our case, it was (13.15 + 11.96)/2 ≈ 12.6 (Table 3).

**Table 3.** Cluster Effects and Effect Modifiers Identified Via the Generalized HTE Approach in the Health and Retirement Student (HRS), *N* = 11,033.

Clusters	CATE (RD scale)	Older adult (≥ 65) (proportion)	Years of education (mean)	Low childhood SES (proportion)
1	0.04	0.51	13.15	0.41
2	0.10	0.83	11.96	0.59

CATE: Individualized conditional average treatment effect; RD: risk difference.

The effect of low kidney function on dementia risk is higher among older adults, those with less years of education and who are more likely to come from a low-income household during childhood.

#### 3.4.3 Estimating subgroup CATEs

In the HRS dataset, using the *generalized HTE approach*, we identified six meaningful subgroups defined by older age, years of education or childhood SES (Table 4): Younger adult (RD: 0.00, 95%CI: [−0.02, 0.03]); older adults (RD: 0.10, 95%CI: [0.08, 0.13]) and p for interaction < 0.001; years of education < 12.6 (Risk Ratio [RR]: 1.59, 95%CI: [1.29, 1.95]); years of education ≥ 12.6 (RR: 2.43, 95%CI: [1.61, 3.65]) and p for interaction < 0.07. Moderate to high childhood SES (RD: 0.04, 95%CI: [0.01, 0.06]); low childhood SES (RD: 0.10, 95%CI: [0.07, 0.13]) and p for interaction < 0.001. Of note, older age was an effect modifier on both absolute and relative scale; years of education was an effect modifier on the relative scale only and childhood SES was an effect modifier on the additive scale only.

**Table 4.** Subgroup CATE on the Additive and Relative Scale Presenting the Effect of Low Kidney Function on Incident Dementia Across Effect Modifiers Identified via the Generalized HTE Approach in the Health and Retirement Student (HRS), *N* = 11,033.

			CATE Risk	*P for*		*P for*
Variables and		Proportion	difference (RD,	*interaction*	CATE Risk ratio	*interaction*
threshold	*N*	(%)	95%CI)	*(RD)*	(RR, 95%CI)	*(RR)*
Total effect	11,033	100	0.07 (0.05, 0.09)	–	1.75 (1.45, 2.11)	–
Younger adult^a^ (<65)	4071	36.90	0.00 (−0.02, 0.03)	0.00	1.07 (0.68, 1.68)	0.02
Older adult^a^ (≥65)	6962	63.10	0.10 (0.08, 0.13)		1.93 (1.55, 2.40)	
Years of education < 12.6	6071	55.03	0.08 (0.05, 0.11)	0.43	1.59 (1.29, 1.95)	0.07
Years of education ≥ 12.6	4962	44.97	0.06 (0.04, 0.08)		2.43 (1.61, 3.65)	
Moderate to high childhood SES	5797	52.54	0.04 (0.01, 0.06)	0.00	1.52 (1.15, 2.02)	0.26
Low childhood SES	5236	47.46	0.10 (0.07, 0.13)		1.89 (1.47, 2.42)	

CI: confidence interval; SES: socio-economic status.

^a^ Age was dichotomized to avoid violation of the positivity assumption.

#### 3.4.4 Shapley values

We estimated the Shapley values by fitting the estimated conditional treatment effect (CATE) on covariates using a random forest model. In particular, the Shapley^87^ values for a feature represent the average marginal contribution of that feature, for a specific observation/individual, to the prediction of CATE; this contribution is obtained by averaging across all possible combinations of features for that specific observation/individual. For instance, for observation 1, being less than 65 contributes the most negatively to the average CATE and low childhood SES contributes the most positively to the average CATE (see eFigure 7). Based on the mean absolute error, the most important feature across all individuals was the feature “older age” (see eFigure 8). The Shapley values were estimated with the help of the R package *iml*. We presented the feature value contribution and the feature importance for the first observation based on the estimated Shapley values in the supplemental materials (eFigure 7, eFigure 8). The top 3 features (using the feature importance) were also those identified as potential effect modifiers using our generalized HTE approach.

## 4 Discussion

### 4.1 Summary and explanation of the main findings

The purpose of this paper was to illustrate, as a proof of concept, that we can use a step-by-step transparent parametric data-adaptive approach (the generalized HTE approach) based on the G-computation algorithm to detect heterogenous subgroups and identify meaningful HTEs. Using real data and simulated data, we were able to identify meaningful HTEs and estimate CATEs by implementing our 7-step data-adaptive approach.

The proposed approach was designed to address three particular challenges when investigating HTEs in epidemiology: (1) the problem of accuracy versus interpretability, (2) the problem of underfitting/overfitting when using parametric modeling, and (3) the problem of type 1 error and joint testing. To solve problems (1) and (2), a regularized transparent supervised machine-learning model such as LASSO, was chosen. The selection of variables to be included in the model was guided by both theory: causal diagrams and background knowledge^32^ as well as by data-adaptive approaches: LASSO.^39^ Lastly, to address problem (3) several strategies were used. First, it employs LASSO which performs variable selection reducing the number of hypotheses tested. Second, cross-validation helps in assessing the model's performance and increases its generalization capability and ultimately reducing the risk of Type I errors. Third, achieving balance in covariates (achieved in step 3 and 7, see eFigure 9 for covariate balance) between candidate subgroups can help prevent the false discovery of HTE subgroups as demonstrated by Rigdon et al.^42^ Fourth, the subgroup effects estimated were only those estimated across identified potential effect modifiers (not across all available covariates as often done in the literature^21,36^)

Our *generalized HTE approach* should in principle be more appealing to epidemiologists interested in explanation and causal inference questions given that: i) it is parametric and transparent, ii) it makes use of statistical interactions to detect heterogeneity, and iii) it explicitly strives to include variables that satisfy the backdoor criterion in an observational setting.

Furthermore, our approach and other methods purporting to identify HTE require sufficient amounts of data to have adequate power to detect heterogeneity. In addition, it may be necessary to evaluate heterogeneity on different scales, that is additive and multiplicative (which, our approach can naturally accommodate)*.* In other words, when HTEs are truly present and there is no bias, failure to detect true HTEs could reflect lack of power or the scale chosen to evaluate heterogeneity. For instance, in our applied example, one variable (years of education) did not modify the effect of low kidney function on dementia risk on the absolute scale; however, it modified such effect on the relative scale. Furthermore, larger sample sizes relative to the number of variables are better for identifying true effect modifiers. In our simulation A3 (*N* = 1000), our approach identified in addition to the two known effect modifiers other variables for which there was little effect modification (eFigure 5). On the other hand, in the larger simulations A1 (*N *= 10,000) and A2 (*N *= 100,000), our approach correctly identified the two known effect modifiers (eFigure 3 and eFigure 4).

### 4.2 Limitations of the current approach, extension and future directions

The approach presented here is a proof of concept and as such does not aim to compare its performance to other HTE-based methods such as causal forest. Future studies should explore the impact of choosing different thresholds for identifying potential effect modifiers as well as the performance of the current approach with several model specifications (e.g. ridge regression), functional forms (e.g. Gaussian, binomial) and different clustering approaches (e.g. k-means, centroid, Ward) against other machine-learning methods including but not limited to causal forest and Bayesian causal forest. The comparison should assess metrics such as root mean square errors/accuracy, bias and coverage in detecting the correct subgroups.

## 5 Conclusion

We illustrated, as a proof of concept, a step-by-step transparent parametric data-adaptive approach (the generalized HTE approach) based on the G-computation algorithm to detect heterogenous subgroups and identify meaningful HTEs. Our generalized approach was able to identify meaningful HTEs and estimate CATE defined by effect modifiers. Future studies should compare the performance of this approach to other HTE methods in terms of bias, coverage and ability to detect subgroups. Our *generalized HTE approach* should in principle be more appealing to epidemiologists interested in explanation/causal inference questions given that it is parametric and transparent, it makes use of statistical interactions to detect heterogeneity, and explicitly strives to include variables that satisfy the backdoor criterion in an observational setting.

## Supplemental Material

sj-docx-1-smm-10.1177_09622802251316969 - Supplemental material for Generalized framework for identifying meaningful heterogenous treatment effects in observational studies: A parametric data-adaptive G-computation approach Supplemental material, sj-docx-1-smm-10.1177_09622802251316969 for Generalized framework for identifying meaningful heterogenous treatment effects in observational studies: A parametric data-adaptive G-computation approach by Roch A. Nianogo, Stephen O'Neill and Kosuke Inoue in Statistical Methods in Medical Research
