# Supplementary material for: Generalized framework for identifying meaningful heterogenous treatment effects in observational studies: A parametric data-adaptive G-computation approach
Source: Stat Methods Med Res. 2025 Feb 24;34(4):648–62. doi: 10.1177/09622802251316969 (PMC12075891; doi:10.1177/09622802251316969)
Supplement: sj-docx-1-smm-10.1177_09622802251316969 - Supplemental material for Generalized framework for identifying meaningful heterogenous treatment effects in observational studies: A parametric data-adaptive G-computation approach [file sj-docx-1-smm-10.1177_09622802251316969.docx]

**SUPPLEMENTAL MATERIALS**

**Generalized Framework for Identifying Meaningful Heterogenous Treatment Effects in Observational Studies: A Parametric Data-Adaptive G-Computation Approach**

Roch A. Nianogo, Stephen O'Neill, Kosuke Inoue

**Table of contents**

[**eSection 1**. Causal structure and data-generating process 2](#_Toc189332614)

[**eSection 2***.* R Code to simulate data (simulation A1) and detect heterogenous treatment effects via the generalized HTE approach can be found at https://github.com/nianogo/generalized_hte 3](#_Toc189332615)

[**eSection 3**. Variable definition 12](#_Toc189332616)

[**eTable 1**. Baseline characteristics in the simulated data A1, N=10,000 4](#_Toc189332623)

[**eTable 2.** Cluster effects and effect modifiers identified via the generalized HTE approach in the simulated data A1 N=10,000 8](#_Toc189332624)

[**eFigure 1.** Histogram of the individualized Conditional Average Treatment Effect (CATE) estimated via the generalized HTE approach in the simulation data A1 (N=10,000) 5](#_Toc189332629)

[**eFigure 2.** Individualized conditional average treatment effects by rank estimated via the generalized HTE approach in the simulated data A1 (N=10,000) 6](#_Toc189332630)

[**eFigure 3.** Variable importance estimated via the generalized HTE approach in the simulated data A1 (N=10,000) 7](#_Toc189332631)

[**eFigure 4.** Variable importance estimated via the generalized HTE approach in the simulated data A2 (N=100,000) 9](#_Toc189332632)

[**eFigure 5.** Variable importance estimated via the generalized HTE approach in the simulated data A3 (N=1,000) 10](#_Toc189332633)

[**eFigure 6**. Individualized conditional average treatment effects by rank estimated via the generalized HTE approach in the Health and Retirement Study (N=11,033) 11](#_Toc189332634)

[**eFigure 7.** Feature value contribution for observation 1 and estimated from the Shapley values in the Health and Retirement Study (N=11,033). 15](#_Toc189332635)

[**eFigure 8.** Feature importance based on the mean absolute error (MAE) for observation 1 and estimated from the Shapley values in the Health and Retirement Study (N=11,033). 16](#_Toc189332636)

[**eFigure 9.** Covariate balance plot showing the absolute mean differences before and after applying the inverse probability of treatment weights (IPW) in the Health and Retirement Study (N=11,033). See **eSection 3** for variable and variable labels. 17](#_Toc189332637)

**eSection 1**. Causal structure and data-generating process

As a proof of concept, we conducted a simulation study to assess the ability of the parametric g-computation approach, *the generalized HTE approach*, to detect HTEs and estimate CATEs. In this simulation, we employed a data-generating process in which we simulated an observational study inspired from the randomized controlled trial simulation study described in Rigdon et al.(Rigdon et al., 2018, *Trials*) We simulated 10,000 individuals (simulation A1) of whom about 30% were taking blood pressure medication to achieve a systolic blood pressure <120 mmHg to reduce cardiovascular outcomes (i.e., intensive blood pressure control). (*Sprint Research Group. A Randomized Trial of Intensive versus Standard Blood-Pressure Control, 2015, New England Journal of Medicine*) To assess the method’s capabilities, we also simulated different sizes (simulation A2, N=100,000 and simulation A3, N=1,000).

Reception of treatment (1= took the treatment [Intensive blood pressure control] and 0=did not take the treatment [standard blood pressure control]) was associated with age, systolic blood pressure, baseline diastolic blood pressure, serum creatinine, estimated glomerular filtration rate (eGFR), statin use, aspirin use, Framingham 10-year risk score, and smoking status (see **eTable 1**). The outcome was 0 in the absence of events and 1 in the presence of events. The average treatment effect was defined as the difference of the potential outcomes, τ = $Y^{1}-Y^{0}$ = $-$0.03. The same 10 baseline covariates affecting treatment reception also affected the outcome. These baseline covariates were simulated as done in Ridgon et al,(Rigdon et al., 2018, *Trials*) that is, uncorrelated with one another. HTEs and regression models were initially estimated from the original data in order to identify corresponding interaction terms needed for the simulation of the new observational data generating process. There were four subgroups defined by aspiring use (participants taking aspirin vs not taking aspirin) and eGFR (<72 vs ≥ 72). Treatment reception, *Tx*, (binary) was simulated as a function of the 10 potential confounders and the outcome of interest, *Y* (binary) was simulated as a function of treatment reception, potential modifiers (eGFR, aspirin) and the 10 potential confounders using Bernoulli trials.

$$Tx = B(n, 1, prob=expit(logit(0.01) + log(0.90)*statin+ log(1.01)*sbp+log(1.01)*dbp+log(1.01)*age+log(1.03)*fram+log(1.1)*black+ log(1.1)*scr+ log(1.1)*smok\_3 +log(1.05)*smok\_2))$$

$Y = B(n, 1, prob=expit(logit(0.01) + log(0.038)*tx+ log(0.592)*I(egfr\geq72) + log(0.453)*aspirin + log(12.00)*tx*I(egfr\geq72) + log(34.70)*tx*aspirin + log(0.543)*I(egfr\geq72)*aspirin + log(0.372)*tx*I(egfr\geq72)*aspirin +log(0.90)*statin+ log(1.01)*sbp+ log(1.01)*dbp+ log(1.01)*age+log(1.03)*fram+log(1.1)*black+ log(1.2)*scr+log(1.05)*smok\_3 +log(1.05)*smok\_2)$

*Log is the natural log, logit(pr) =log (odds(pr)) = log(pr/(1-pr)) where pr is the probability and expit() is the inverse logit function which returns a probability between 1 or 0.*

*Tx, treatment reception, Y, outcome, sbp, systolic blood pressure, dbp, diastolic blood pressure, black, Black race, scr, serum creatinine, fram, Framingham 10-year risk score, smok_3, dummy variable for current smoker, smok_2, dummy variable for former smoker, I(egfr ≥ 72), dummy variable for eGFR ≥ 72.*

**eSection 2***.* R Code to simulate data (simulation A1) and detect heterogenous treatment effects via the generalized HTE approach can be found at <https://github.com/nianogo/generalized_hte>

**eTable 1**. Baseline characteristics in the simulated data A1, N=10,000

|  | Treatment Assignment | | |
| --- | --- | --- | --- |
| Variable | Overall, N = 10,000 | No treatment,  N = 6,944 | Treatment,  N = 3,056 |
| **Age in years, Mean (SD)** | 68 (10) | 68 (10) | 69 (10) |
| **Black race, n (%)** | 3,040 (30%) | 2,084 (30%) | 956 (31%) |
| **Systolic blood pressure, Mean (SD)** | 140 (15) | 139 (15) | 141 (15) |
| **Diastolic blood pressure, Mean (SD)** | 78 (12) | 78 (12) | 79 (12) |
| **Serum Creatinine, Mean (SD)** | 1 (0) | 1 (0) | 1 (0) |
| **Estimated Glomerular Filtration Rate, Mean (SD)** | 72 (20) | 72 (20) | 73 (20) |
| **Statin use, n (%)** | 4,340 (43%) | 3,094 (45%) | 1,246 (41%) |
| **Aspirin use, n (%)** | 5,117 (51%) | 3,540 (51%) | 1,577 (52%) |
| **Framingham score risk, Mean (SD)** | 25 (12) | 24 (12) | 28 (12) |
| **Smoking status, n (%)** |  |  |  |
| Never smoker | 4,400 (44%) | 3,074 (44%) | 1,326 (43%) |
| Former smoker | 4,168 (42%) | 2,905 (42%) | 1,263 (41%) |
| Current smoker | 1,432 (14%) | 965 (14%) | 467 (15%) |
| **Y (cardiovascular events), n (%)** | 1,808 (18%) | 1,281 (18%) | 527 (17%) |

SD: Standard deviation; Y is the outcome—the presence of cardiovascular events


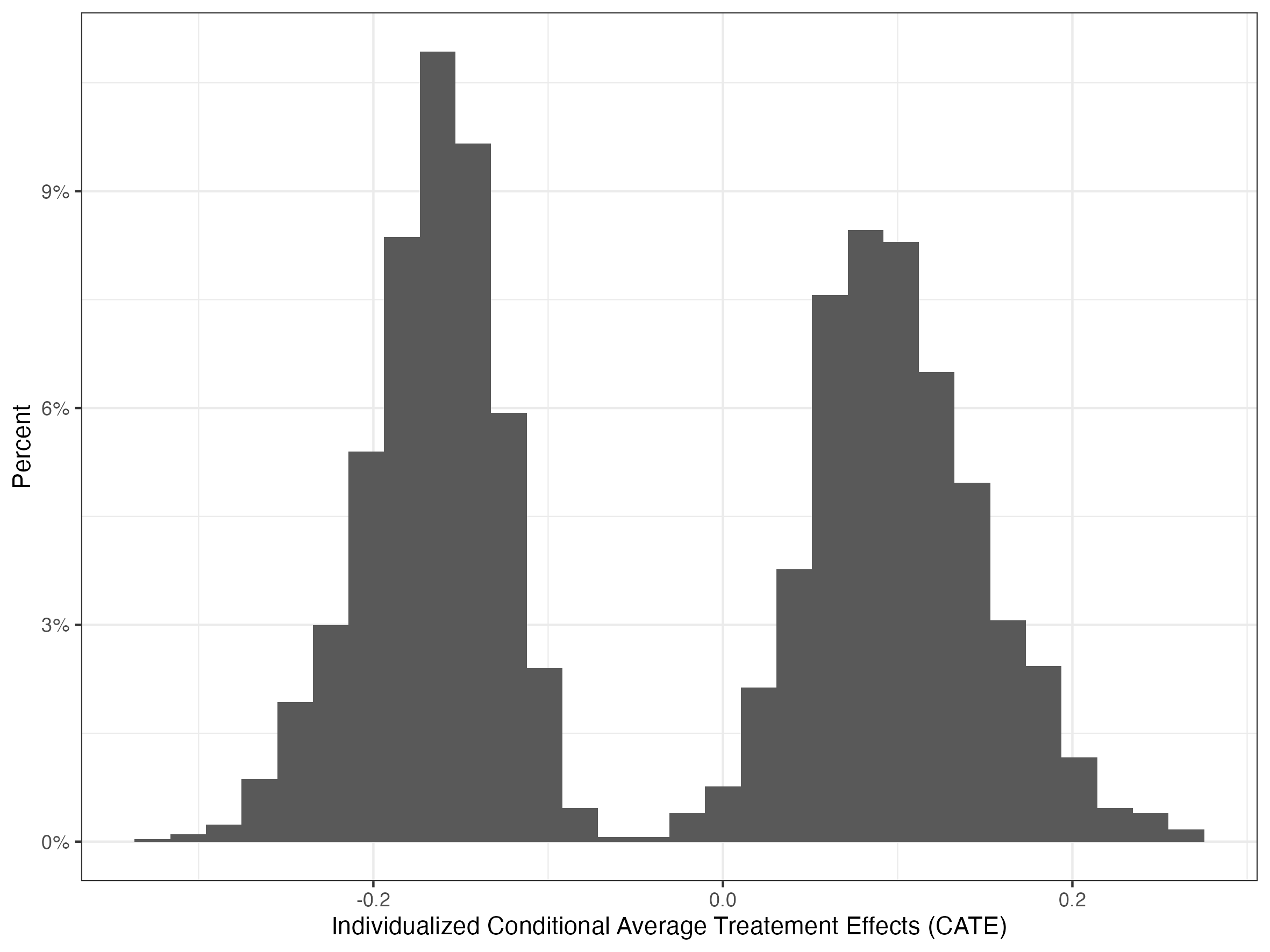


**eFigure 1.** Histogram of the individualized Conditional Average Treatment Effect (CATE) estimated via the generalized HTE approach in the simulation data A1 (N=10,000)


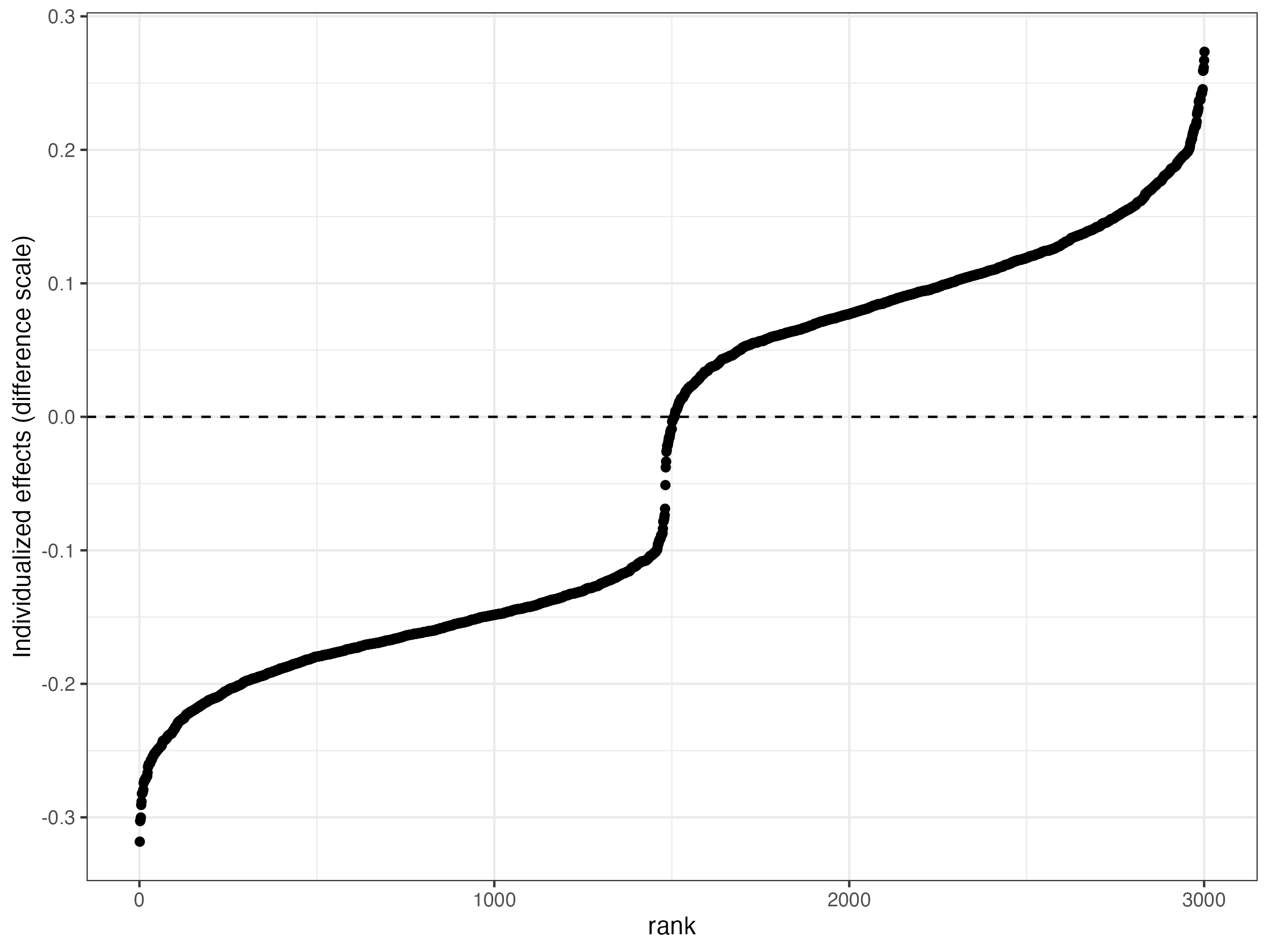


**eFigure 2.** Individualized conditional average treatment effects by rank estimated via the generalized HTE approach in the simulated data A1 (N=10,000)


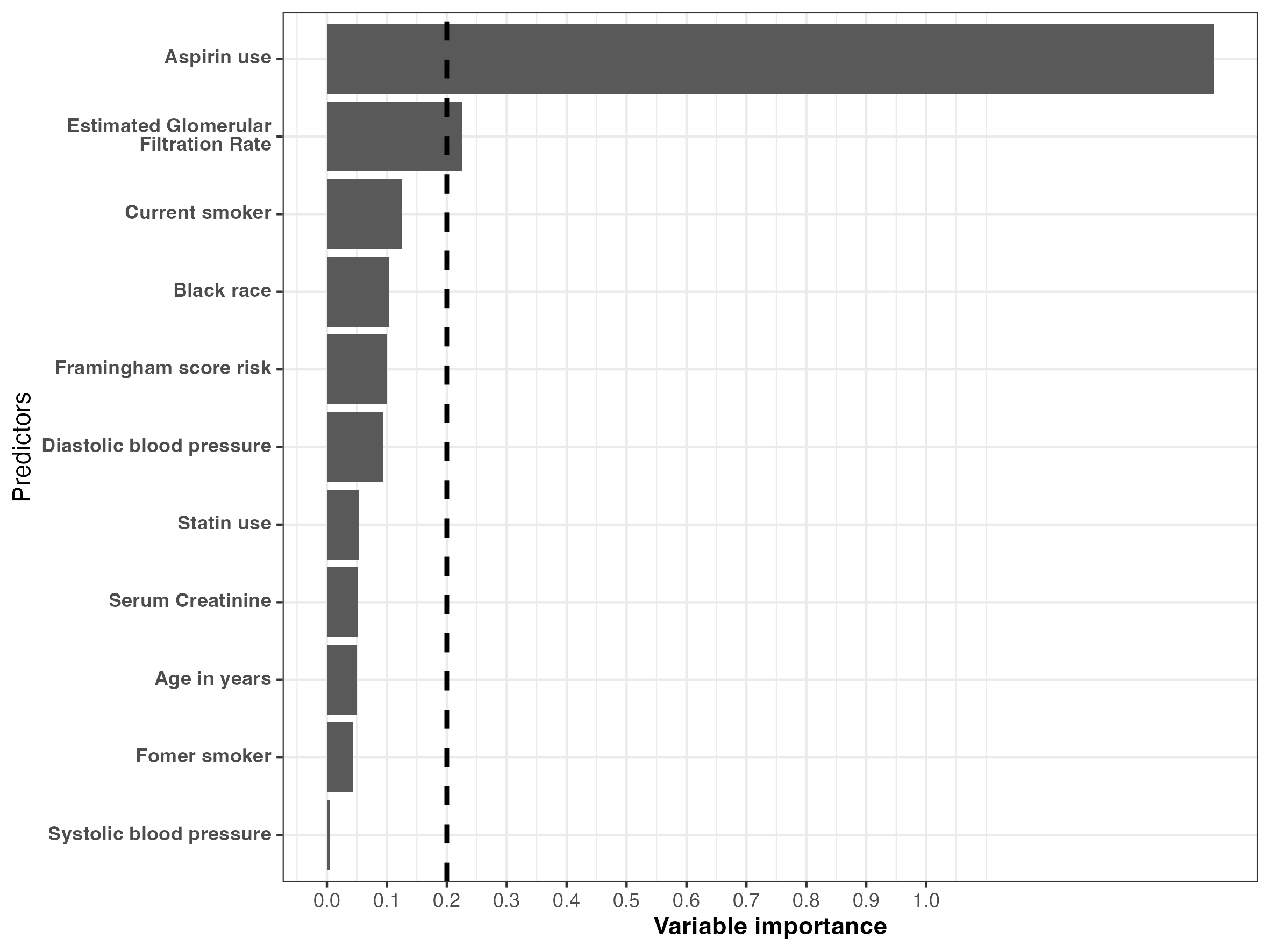


**eFigure 3.** Variable importance estimated via the generalized HTE approach in the simulated data A1 (N=10,000)

**eTable 2.** Cluster effects and effect modifiers identified via the generalized HTE approach in the simulated data A1 N=10,000

| Clusters | CATE  (RD scale) | eGFR  (mean) | Aspirin use  (proportion) |
| --- | --- | --- | --- |
| 1 | 0.10 | 75.31 | 0.97 |
| 2 | -0.11 | 70.74 | 0.23 |

CATE, Individualized conditional average treatment effect. RD, risk difference, eGFR, estimated glomerular filtration rate

*The effect of treatment on incident atherosclerosis events is more beneficial (larger) for those who have lower eGFR and less likely to be taking aspirin and harmful for those who have a higher eGFR and more likely to be taking aspirin*


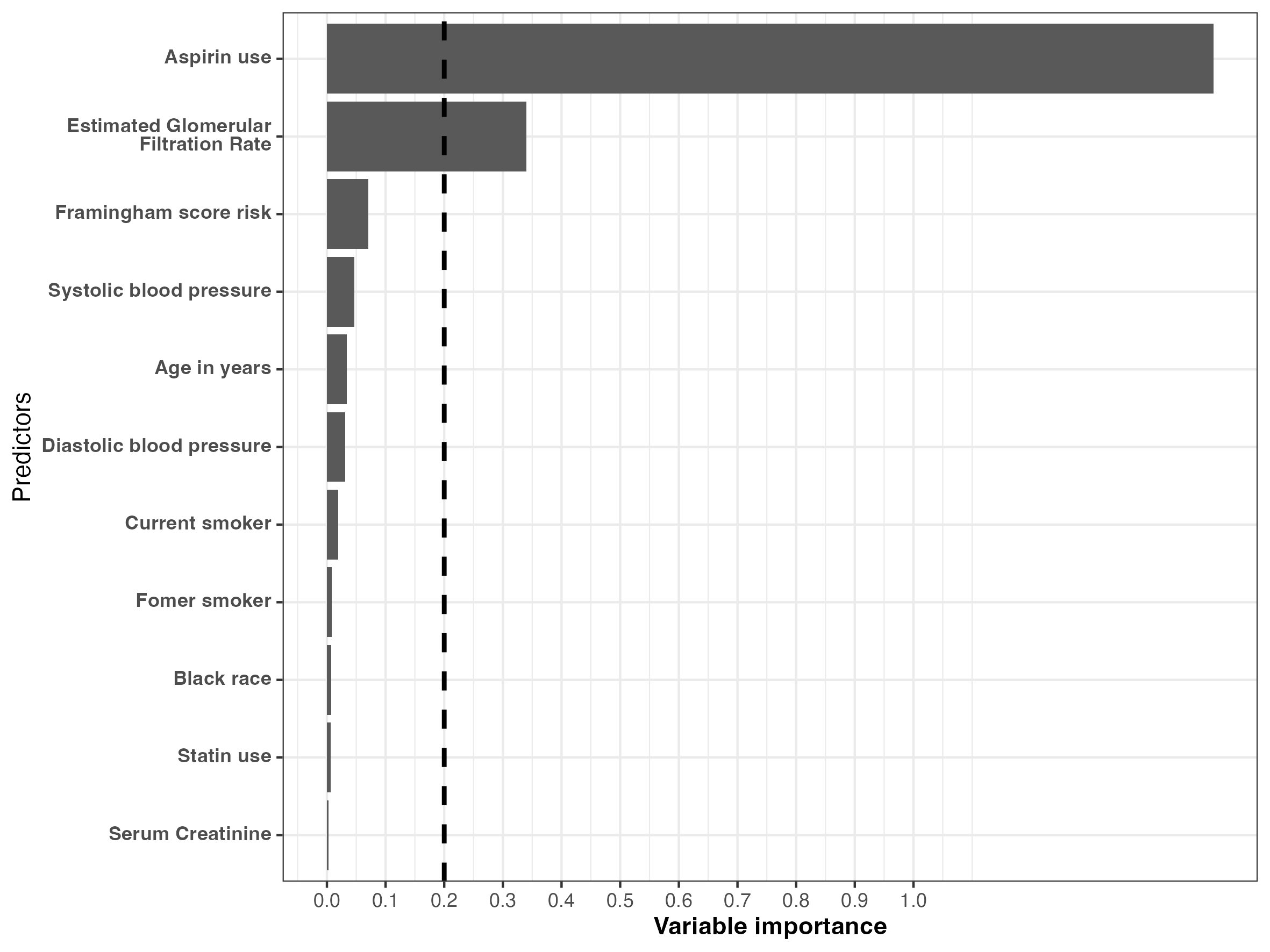


**eFigure 4.** Variable importance estimated via the generalized HTE approach in the simulated data A2 (N=100,000)


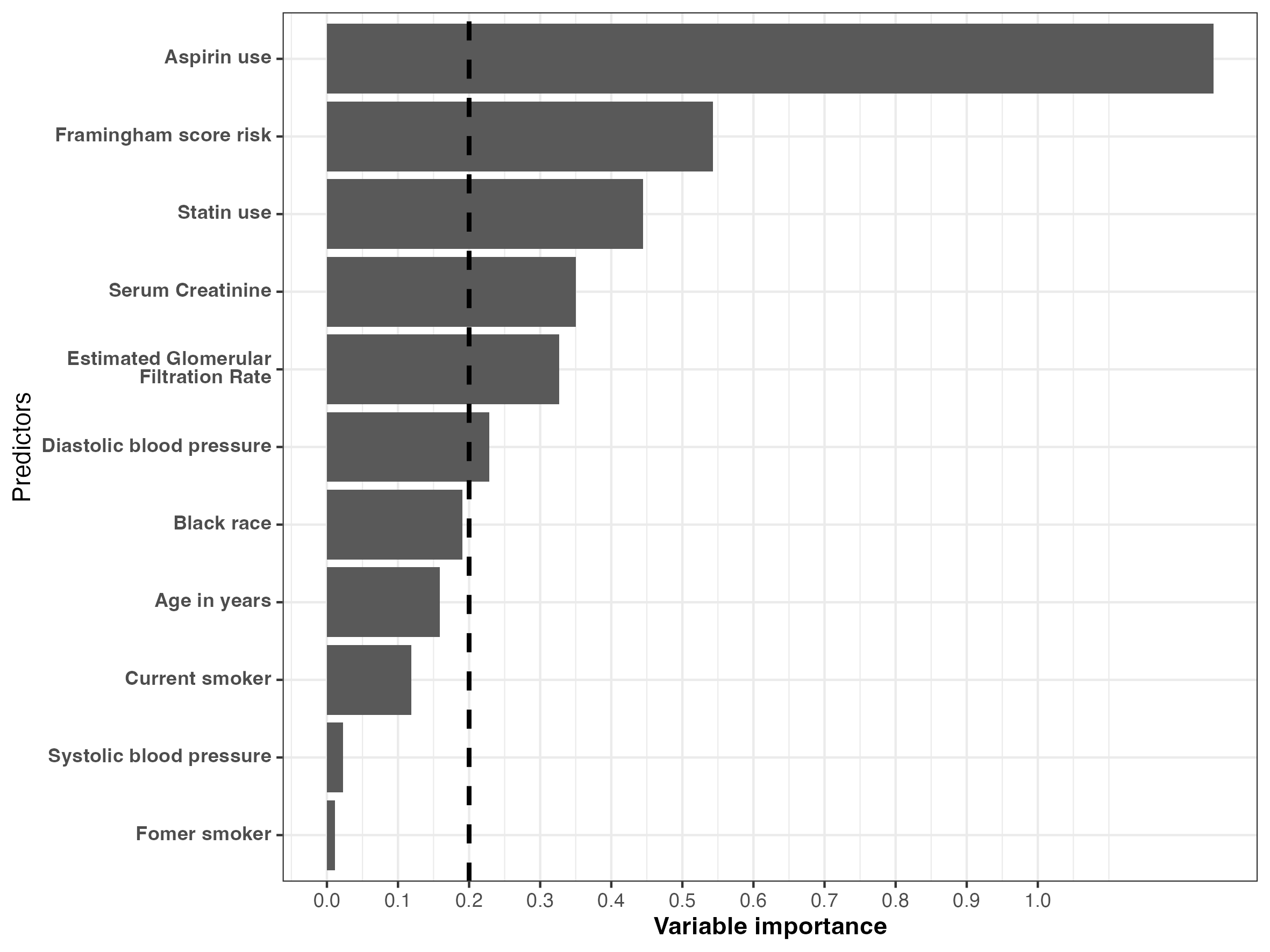


**eFigure 5.** Variable importance estimated via the generalized HTE approach in the simulated data A3 (N=1,000)


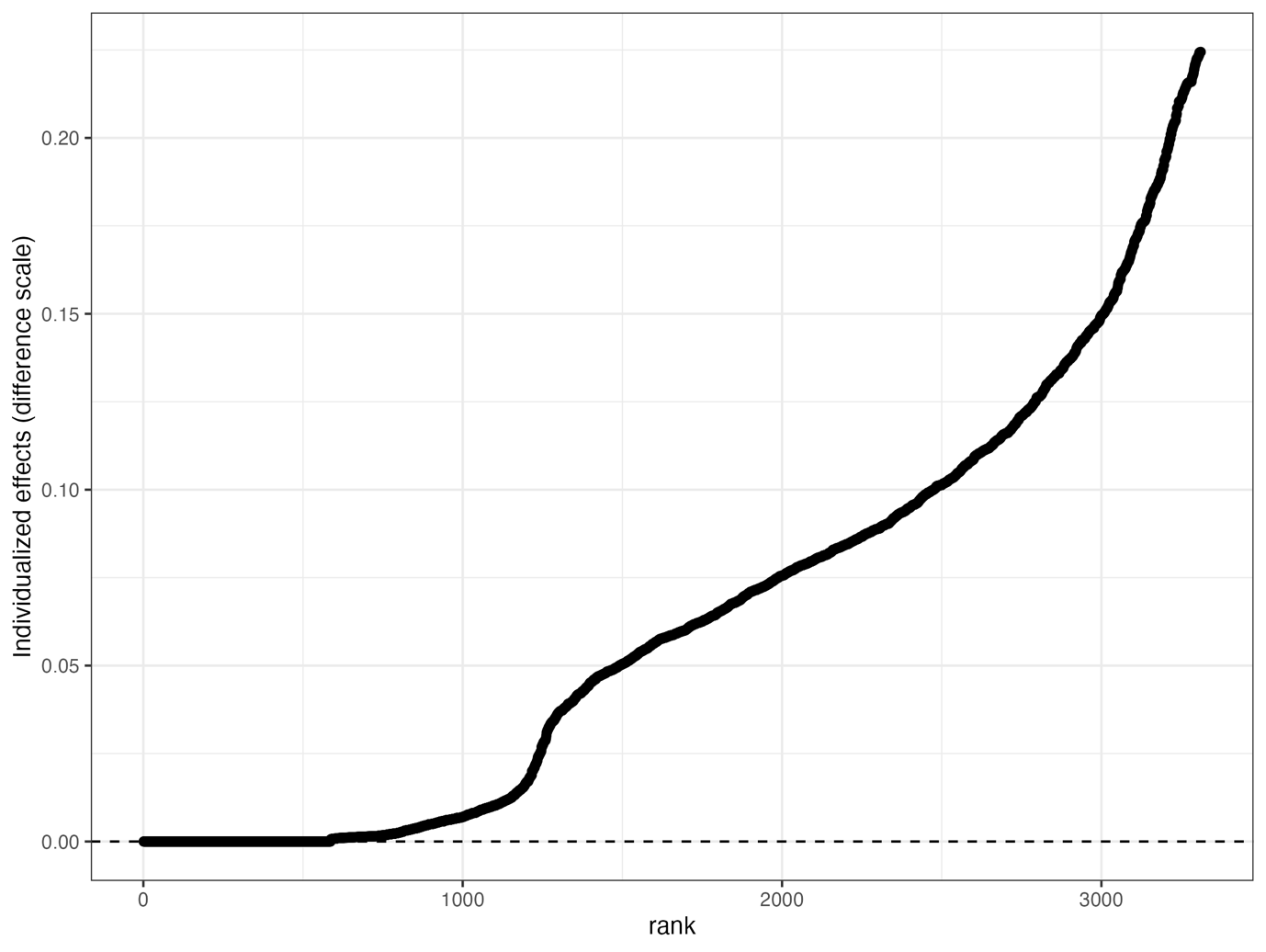


**eFigure 6**. Individualized conditional average treatment effects by rank estimated via the generalized HTE approach in the Health and Retirement Study (N=11,033)

**eSection 3**. Variable definition

| **Variable names** | **Variable labels** | **Variable type:**  **Continuous (units) or Binary** | **How the variable was measured and defined** |
| --- | --- | --- | --- |
| low_egfr or tx | Low estimated glomerular filtration (eGFR) | Binary (yes vs no) | Estimated Glomerular Filtration (eGFR) based on measured serum Cystatin C using the 2012 epidemiologic chronic kidney disease equation (2012 CKD-EPI-cystatin). This variable was dichotomized as low (eGFR < 90 mL/min/1.73 m^2^) vs normal (eGFR >= 90 mL/min/1.73 m^2^). |
| dementia or y | Incident dementia | Binary (dementia vs not dementia) | Incident dementia was defined using a 27-point cognitive score (range 0-27) and classified using the Langa-Weir classification as 0-6 as dementia, 7-11 as Cognitively Impaired not Dementia (CIND), and 12-27 as normal (higher scores are better). For this illustration, we compared dementia vs no dementia (which included CIND and normal). After removing prevalent dementia cases at baseline (2006), other dementia cases that occurred during follow-up and up to the end of follow-up (2020) was considered and incident case. |
| older_age | Older age (Age>=65) | Binary (Age>=65 vs Age < 65) | Age in years was dichotomized to avoid violation of the positivity assumption |
| edu_yrs | Participant years of education | Continuous (years) | Number of years of education |
| low_child_ses | Low child SES | Binary (low vs high) | Measured by the participant’s father’s and mother’s highest education. Low childhood socio-economic status (SES) was defined as having both father and mother’s education to be less than 12 years, and high childhood SES, otherwise |
| non_white | Non-NH Whites participants | Binary (Non-NH Whites vs NH Whites) | Self-reported race/ethnicity was dichotomized as Non-NH Whites (i.e. minority, which included NH Black, Hispanic, and Others) vs NH Whites. NH means Not Hispanic |
| exercise | Exercise (MET>=35) | Binary (high: MET >=35 vs low: MET < 35). | This variable was constructed by summing the metabolic equivalent of tasks [MET] of participant’s physical activity level. The MET measures the amount of energy expenditure as a function of cumulative physical activities. Exercise or high level of physical activity was defined as having a MET score >=35. A low level of physical activity (or no exercise) was defined as having a MET score < 35. |
| bmi_cont | Body mass index (BMI), kg/m^2^ | Continuous (kg/m^2^) | Calculated by dividing measured weight in kilogram by measured height squared |
| sbp_cont | Systolic Blood Pressure (mmHg) | Continuous (mmHg) | Measured systolic blood pressure |
| drink_number | Number of alcoholic drinks in last 3 months | Continuous (number of drinks) | Self-reported number of alcoholic drinks in last 3 months |
| low_income | Income < 130 of the FPL | Binary (Low-income: Income < 130 of the FPL vs moderate to high income: >= 130 of the FPL) | Income was dichotomized as low-income: Income < 130 of the FPL and moderate to high income: >= 130 of the FPL |
| hba1c_cont | HBA1c (%): NHANES Equivalent | Continuous (%) | Measured hemoglobin A1c (%) |
| married | Married | Binary (Married vs not married) | Self-reported marital status. This variable was dichotomized as married vs not married (which included separated, divorced, widowed, or never married) |
| apoe4 | APOE 4 | Binary (yes vs no) | Assessed using whether the participant tested positive for the Apolipoprotein E4 (apoE4) gene |
| cesd_score | CESD/Depression Score | Continuous (score) | Measured using the abbreviated 8-item Center for Epidemiological Studies Depression [CESD] scale. This scale is used to assess depressive symptoms. The final used index score was estimated as an average of the responses across relevant items. Higher scores indicated greater depressive symptoms. |
| state_anger | State Anger Index | Continuous (score) | Measured using the Spielberger Anger Expression Scale (STAX). This scale can be used to assess state anger (anger-out) which refers to a more temporary angry reaction usually expressed through behavior. The final used index score was estimated as an average of the responses across relevant items. Higher scores indicated greater state anxiety. |
| trait_anger | Trait Anger Index | Continuous (score) | Measured using the Spielberger Anger Expression Scale (STAX). This scale can be to assess trait anger (anger-in) which refers to a more stable predisposition to respond to a range of situations with an angry response. The final used index score was estimated as an average of the responses across relevant items. Higher scores indicated greater trait anxiety. |
| anxiety | Anxiety Index | Continuous (score) | Measured using the 5-item Beck Anxiety Inventory. The final used index score was estimated as an average of the responses across items. Higher scores indicated greater anxiety. |
| crp_cont | C-reactive Protein (mg/L); NHANES Equivalent | Continuous (mg/L) | Measured high-sensitivity C-Reactive protein (mg/L) |
| waist_cont | Waist Circumference (cm) | Continuous (cm) | Measured waist circumference (cm) |
| pulse_cont | Pulse (Beats per Minute) | Continuous (beats per minute) | Measured pulse (beats per minute) |
| no_smoking | Never smoked | Binary (never vs ever smoked) | The smoking variable was categorized as “never smoked” vs “ever smoked”—which included those who were current or former smoker |
| female | Female sex | Binary (1=female sex/gender, 0=Male sex/gender) | Self-reported sex/gender |
| hdl_cont | High-density lipoprotein (HDL) (mg/dL); NHANES Equivalent | Continuous (mg/dL) | Measured high-density lipoprotein (HDL) cholesterol measured from dried blood spot samples |
| *Tx is the treatment/exposure [low eGFR vs Normal]; Y is the outcome (dementia [yes vs no]); SES: Socio-economic status; MET: Metabolic Equivalent of task; CESD: Center for Epidemiologic Studies Depression Scale; APOE4: Apolipoprotein E4; HbA1c: Hemoglobin A1c; NHANES: National Health and Nutrition Examination Survey; eGFR: estimated glomerular filtration rate; FPL: Federal poverty level, HDL: High-density lipoprotein.*  *The exposure and covariate variables were assessed at baseline, while incident dementia was assessed throughout the follow-up after baseline. Missing data was dealt with by multiple imputation assuming the data are missing at random. In this illustration, we used the first imputed dataset with complete data.* | | | |


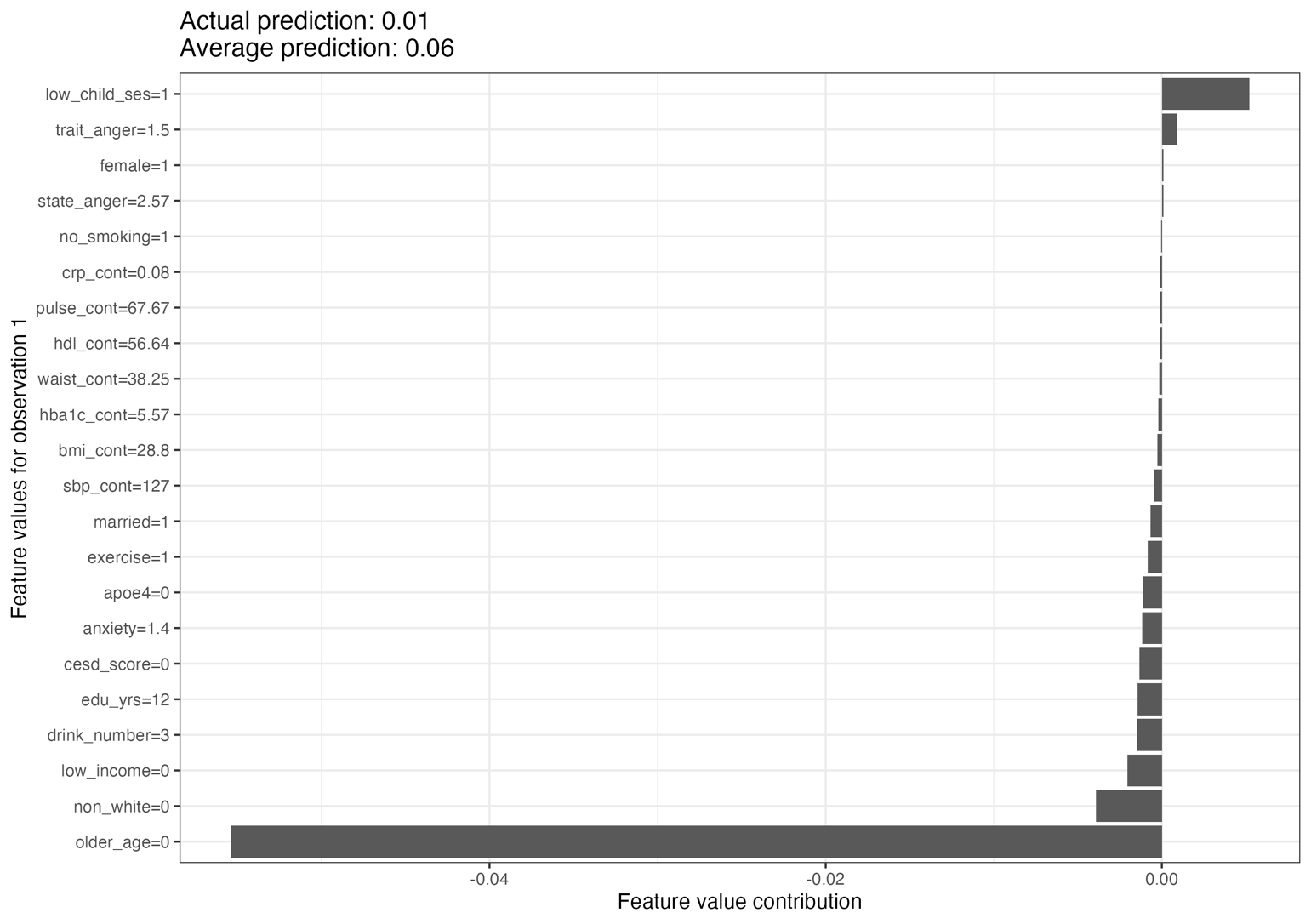


**eFigure 7.** Feature value contribution for observation 1 and estimated from the Shapley values in the Health and Retirement Study (N=11,033).

The estimated individualized conditional average treatment effect was fit on covariates using a random forest model. The Shapley values were subsequently estimated using the package IML in R. See **eSection 3** for variable and variable labels.


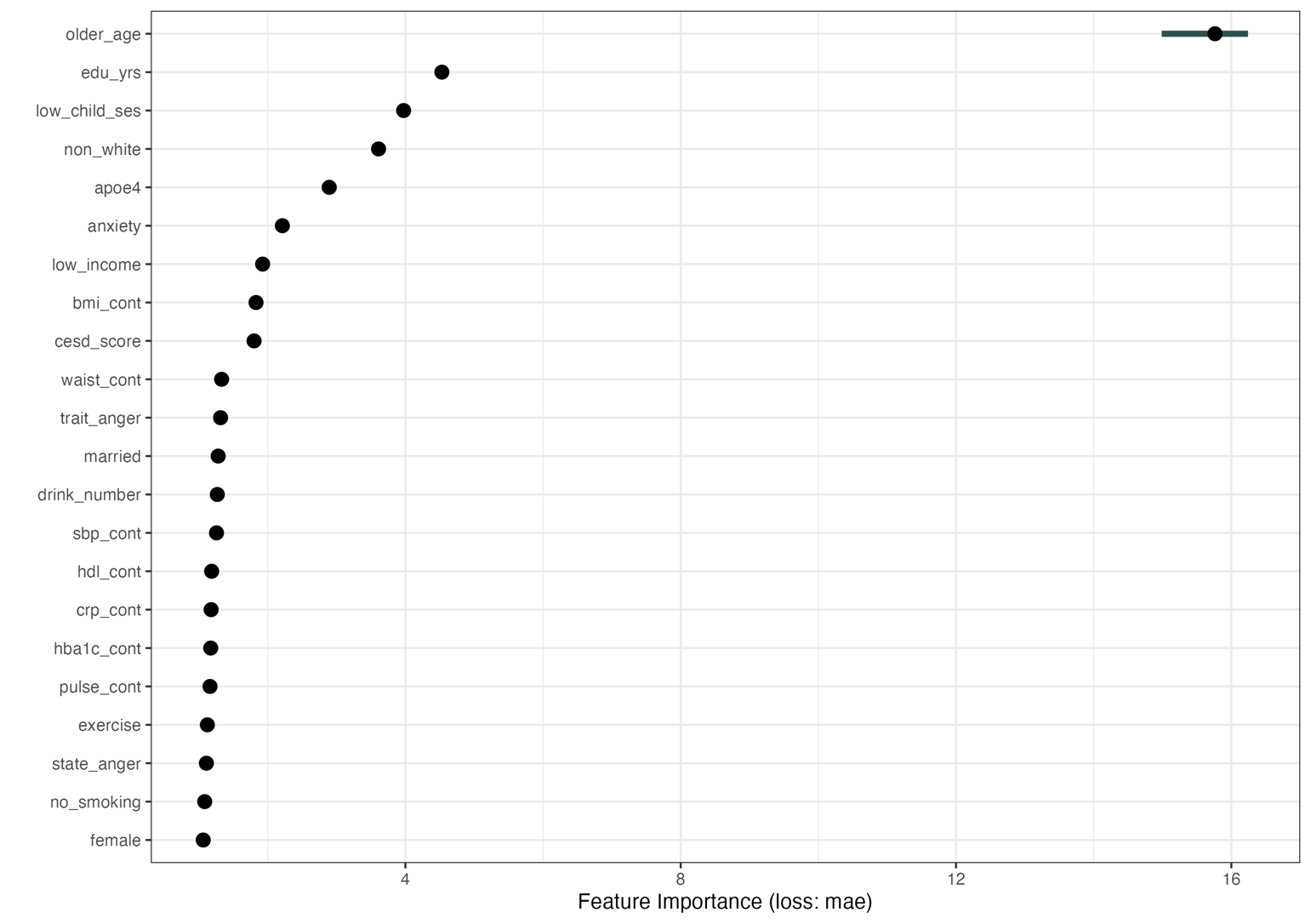


**eFigure 8.** Feature importance based on the mean absolute error (MAE) for observation 1 and estimated from the Shapley values in the Health and Retirement Study (N=11,033).

The estimated individualized conditional average treatment effect was fit on covariates using a random forest model. The Shapley values were subsequently estimated using the package IML in R. See **eSection 3** for variable and variable labels.


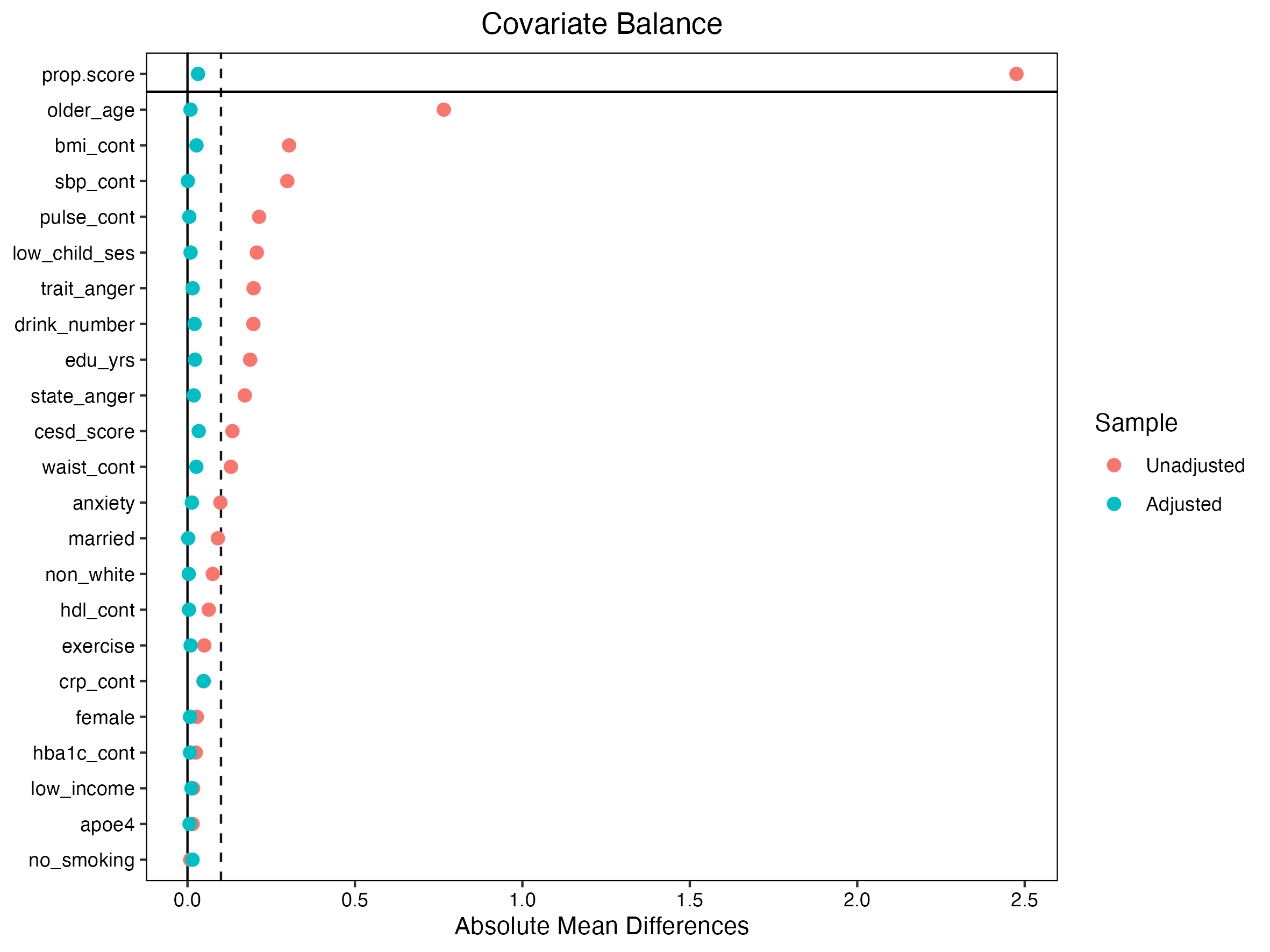


**eFigure 9.** Covariate balance plot showing the absolute mean differences before and after applying the inverse probability of treatment weights (IPW) in the Health and Retirement Study (N=11,033). See **eSection 3** for variable and variable labels.
